# Daratumumab, lenalidomide, and dexamethasone in East Asian patients with relapsed or refractory multiple myeloma: subgroup analyses of the phase 3 POLLUX study

**DOI:** 10.1038/s41408-018-0071-x

**Published:** 2018-05-01

**Authors:** Kenshi Suzuki, Meletios A. Dimopoulos, Naoki Takezako, Shinichiro Okamoto, Atsushi Shinagawa, Morio Matsumoto, Hiroshi Kosugi, Sung-Soo Yoon, Shang-Yi Huang, Xiang Qin, Ming Qi, Shinsuke Iida

**Affiliations:** 1 Department of Hematology, Japanese Red Cross Medical Center, Tokyo, Japan; 2 National and Kapodistrian University of Athens, Athens, Greece; 3 Department of Hematology, National Hospital Organization Disaster Medical Center of Japan, Tachikawa, Japan; 4 Keio University Hospital, Tokyo, Japan; 5 Hitachi General Hospital, Hitachi, Japan; 6 Department of Hematology, National Hospital Organization Shibukawa Medical Center, Shibukawa, Japan; 7 Department of Hematology, Ogaki Municipal Hospital, Ogaki, Japan; 8 Seoul National University Hospital, Seoul, Korea; 9 National Taiwan University Hospital, Taipei, Taiwan; 10 Janssen Research & Development, LLC, Spring House, PA USA; 11 Nagoya City University Graduate School of Medical Sciences, Nagoya, Japan

## Abstract

In the phase 3 POLLUX study, daratumumab plus lenalidomide and dexamethasone (DRd) significantly reduced the risk of progression/death and induced deeper responses vs. lenalidomide and dexamethasone alone (Rd) in patients with relapsed/refractory multiple myeloma (RRMM). We report a subgroup analysis of East Asian (Japanese, Korean, and Taiwanese) patients from POLLUX based on a longer follow-up of 24.7 months. Median progression-free survival was not reached (NR) for DRd vs. 13.8 months for Rd (hazard ratio [HR], 0.42; 95% confidence interval [CI], 0.23–0.76), and overall response rates were higher for DRd vs. Rd (90.2 vs. 72.1%). DRd extended the median duration of response vs. Rd (NR vs. 20.2 months), and minimal residual disease–negative rates at the 10^–5^ sensitivity threshold were 21.2 vs. 9.1% for DRd vs. Rd. No new safety signals were observed. Similar efficacy and safety were observed in the smaller subgroup of Japanese patients treated with DRd vs. Rd. These results demonstrate favorable efficacy and safety of DRd vs. Rd in East Asian patients and also in the Japanese-only patient subgroup that are consistent with findings in the overall patient population of POLLUX.

## Introduction

Daratumumab, a human IgG1κ monoclonal antibody that targets the cell surface protein CD38, demonstrates on-tumor and immunomodulatory mechanisms of action in multiple myeloma (MM)^1–6^. Daratumumab exerts its antimyeloma activity via multiple mechanisms, including direct apoptosis induction, complement-dependent cytotoxicity, antibody-dependent cell-mediated cytotoxicity, antibody-dependent cellular phagocytosis, and modulation of the enzymatic activities of CD38^1–4,6^. Daratumumab also binds CD38 on immunosuppressive regulatory cells, triggering the expansion and activation of cytotoxic T-cells and elevation in T-cell clonality, which may provide additional antimyeloma effects^5^. Based on the results of single-agent and combination therapy studies, daratumumab was approved as a monotherapy and in combination with standard of care regimens across many countries in patients with relapsed and/or refractory multiple myeloma (RRMM)^7–12^. Recently, daratumumab in combination with lenalidomide and dexamethasone (Rd) or bortezomib and dexamethasone was approved for treatment of adults with RRMM in Japan^13^.

The POLLUX study compared the efficacy and safety of daratumumab in combination with Rd (DRd) vs. Rd alone in patients with RRMM who received at least one prior line of therapy^9^. The addition of daratumumab to Rd significantly prolonged progression-free survival (PFS; median, not reached (NR) vs. 18.4 months; hazard ratio [HR], 0.37; 95% confidence interval [CI], 0.27–0.52; *P* < 0.001) and increased the overall response rate (ORR; 92.9 vs. 76.4%; *P* < 0.001). A novel aspect of the POLLUX study was a prospective analysis of minimal residual disease (MRD) in RRMM patients. At a sensitivity threshold of one tumor cell per 100,000 white cells (10^–5^), 22.4% of patients treated with DRd were below this threshold compared with 4.6% in the control group, highlighting the very deep responses induced by daratumumab-based treatment^9^. The adverse event profile was clinically manageable and was consistent with those of daratumumab and Rd alone^9^.

The efficacy and safety profiles of targeted anticancer therapies in East Asian patients may differ from those of the overall study populations in clinical trials^14,15^. To understand the impact of DRd vs. Rd in the East Asian patient population, we performed subanalyses of POLLUX data to evaluate the efficacy and safety of DRd vs. Rd in East Asian (Japanese, Korean, and Taiwanese) patients as well as in only Japanese patients.

## Patients and methods

### Patients

A total of 96 East Asian patients from the phase 3 POLLUX clinical trial (ClinicalTrials.gov identifier: NCT02076009) were included in this analysis. A separate subanalysis of the 36 Japanese patients alone was also conducted. Study design, patient eligibility, treatment schedule, and statistical analyses were previously published^9^. In brief, eligible patients had documented MM, measurable disease at screening, and progressive disease during or after receiving their last regimen, and had received and responded to one or more previous lines of therapy. Measurable disease was defined according to serum or urine M-protein levels or serum-free light chain levels and abnormal serum immunoglobulin-free light chain ratios (kappa:lambda light chains). Progressive disease was defined according to International Myeloma Working Group (IMWG) criteria^16^. Patients with lenalidomide-refractory disease or who had discontinued previous lenalidomide treatment due to adverse events were excluded from the study.

### Dosing

Patients were randomized (1:1) to receive 28-day cycles of DRd or Rd alone until disease progression, unacceptable toxicity, withdrawal of consent, or death. Daratumumab was administered at 16 mg/kg intravenously once weekly during Cycles 1 and 2, every 2 weeks during Cycles 3–6, and every 4 weeks thereafter. Patients with creatinine clearance >60 ml/min received 25 mg lenalidomide orally on Days 1–21 of each cycle; patients with creatinine clearance of 30–60 ml/min received 10 mg lenalidomide daily. Dexamethasone was administered at 40 mg weekly. Patients in the DRd group received a split dose of dexamethasone on weeks when daratumumab was administered: 20 mg of dexamethasone before the daratumumab infusion and 20 mg the day after the daratumumab infusion. Patients aged >75 years or with a body mass index <18.5 kg/m^2^ received a reduced dose of dexamethasone (20 mg) weekly at the physician’s discretion.

### Evaluation and statistical analyses

Responses were evaluated based on IMWG criteria^16,17^. The Kaplan–Meier method was used to evaluate PFS, overall survival (OS), and duration of response. A Cox regression model was used to estimate 95% CIs. MRD status was assessed by next-generation sequencing (NGS) using bone marrow obtained from patients who had a suspected complete response (CR) and was measured at three sensitivity thresholds: 10^–4^, 10^–5^, and 10^–6^, corresponding to one tumor cell per 10^4^, 10^5^, and 10^6^ white blood cells, respectively. The MRD-negative rate was defined as the proportion of subjects who achieved MRD negativity at any time after their first dose of daratumumab. In our MRD analysis, patients in the intent-to-treat population who did not undergo MRD assessment were considered to be MRD-positive. Cytogenetic risk status was assessed by fluorescence in situ hybridization or karyotype testing. Patients were considered high risk if they had at least one of the following cytogenetic abnormalities: del(17p), t(4;14), or t(14;16); patients lacking all three of these abnormalities were considered standard risk.

## Results

### Patients and treatment

Patients in POLLUX were randomized between June 2014 and July 2015, and the clinical cutoff date for this analysis was 7 March 2017. East Asian patients comprised 16.9% (96/569) of the overall population of patients in POLLUX, with Japanese patients constituting 6.3% (36/569) of the POLLUX study population. Fifty-two East Asian (21 Japanese) patients were randomized to the DRd group, and 44 East Asian (15 Japanese) patients were randomized to the Rd group (Table 1). The median (range) age was 64 (34–85) years for East Asian patients and 68 (45–81) years for Japanese patients. The median (range) time since diagnosis was 3.3 (0.8–27.0) years for East Asian patients and 3.0 (0.9–27.0) years for Japanese patients.

**Table 1:** Demographics and baseline characteristics

	East Asian patients^a^		Japanese patients	
	DRd (*n* = 52)	Rd (*n* = 44)	DRd (*n* = 21)	Rd (*n* = 15)
Median (range) age, years	64 (34–80)	65 (44–85)	68 (45–80)	67 (50–81)
<65, *n* (%)	28 (53.8)	21 (47.7)	6 (28.6)	4 (26.7)
65–74, *n* (%)	18 (34.6)	19 (43.2)	12 (57.1)	9 (60.0)
≥75, *n* (%)	6 (11.5)	4 (9.1)	3 (14.3)	2 (13.3)
Female/male sex, %	50.0/50.0	38.6/61.4	47.6/52.4	40.0/60.0
ECOG score, *n* (%)				
0	25 (48.1)	21 (47.7)	14 (66.7)	10 (66.7)
1	25 (48.1)	22 (50.0)	7 (33.3)	4 (26.7)
2	2 (3.8)	1 (2.3)	0 (0.0)	1 (6.7)
Risk stratification^b^, *n*	50	42	20	15
Standard risk, *n* (%)	46 (92.0)	35 (83.3)	17 (85.0)	10 (66.7)
High risk, *n* (%)	4 (8.0)	7 (16.7)	3 (15.0)	5 (33.3)
del(17p)	3 (6.0)	3 (7.1)	3 (15.0)	3 (20.0)
t(4;14)	1 (2.0)	6 (14.3)	0 (0.0)	4 (26.7)
t(14;16)	0 (0.0)	1 (2.4)	0 (0.0)	1 (6.7)
Isotype, *n* (%)				
IgG	31 (59.6)	23 (52.3)	16 (76.2)	8 (53.3)
IgA	9 (17.3)	13 (29.5)	0 (0.0)	4 (26.7)
IgM	1 (1.9)	0 (0.0)	1 (4.8)	0 (0.0)
IgD	2 (3.8)	2 (4.5)	0 (0.0)	1 (6.7)
IgE	0 (0.0)	0 (0.0)	0 (0.0)	0 (0.0)
Light chain	7 (13.5)	5 (11.4)	3 (14.3)	1 (6.7)
Kappa	4 (7.7)	3 (6.8)	1 (4.8)	1 (6.7)
Lambda	3 (5.8)	2 (4.5)	2 (9.5)	0 (0.0)
Biclonal	0 (0.0)	0 (0.0)	0 (0.0)	0 (0.0)
Negative immunofixation	2 (3.8)	1 (2.3)	1 (4.8)	1 (6.7)
Median (range) time since diagnosis, years	3.9 (0.8–27.0)	2.6 (0.9–13.5)	3.4 (0.9–27.0)	3.0 (0.9–13.5)
Median (range) number of prior lines	2.0 (1–6)	1.5 (1–6)	1.0 (1–6)	1.0 (1–6)
1 prior line, *n* (%)	25 (48.1)	22 (50.0)	13 (61.9)	8 (53.3)
2 prior lines, *n* (%)	17 (32.7)	11 (25.0)	2 (9.5)	6 (40.0)
3 prior lines, *n* (%)	6 (11.5)	8 (18.2)	3 (14.3)	0 (0.0)
>3 prior lines, *n* (%)	4 (7.7)	3 (6.8)	3 (14.3)	1 (6.7)
Prior ASCT, *n* (%)	36 (69.2)	24 (54.5)	12 (57.1)	6 (40.0)
Prior PI, *n* (%)	43 (82.7)	33 (75.0)	17 (81.0)	13 (86.7)
Bortezomib	43 (82.7)	33 (75.0)	17 (81.0)	13 (86.7)
Carfilzomib	0 (0.0)	0 (0.0)	0 (0.0)	0 (0.0)
Ixazomib	0 (0.0)	1 (2.3)	0 (0.0)	1 (6.7)
Prior IMiD, *n* (%)	28 (53.8)	29 (65.9)	7 (33.3)	9 (60.0)
Lenalidomide	3 (5.8)	2 (4.5)	3 (14.3)	2 (13.3)
Pomalidomide	0 (0.0)	0 (0.0)	0 (0.0)	0 (0.0)
Thalidomide	28 (53.8)	28 (63.6)	7 (33.3)	8 (53.3)
Prior corticosteroid, *n* (%)	52 (100.0)	44 (100.0)	21 (100.0)	15 (100.0)
Dexamethasone	46 (88.5)	37 (84.1)	16 (76.2)	13 (86.7)
Prednisone	19 (36.5)	18 (40.9)	8 (38.1)	7 (46.7)
Prior alkylating agent, *n* (%)	50 (96.2)	39 (88.6)	19 (90.5)	14 (93.3)
Prior PI+IMiD, *n* (%)	22 (42.3)	20 (45.5)	5 (23.8)	7 (46.7)
Refractory to, *n* (%)				
Last line of therapy	19 (36.5)	24 (54.5)	8 (38.1)	8 (53.3)
Bortezomib	14 (26.9)	16 (36.4)	7 (33.3)	7 (46.7)

*DRd* daratumumab/lenalidomide/dexamethasone, *Rd* lenalidomide/dexamethasone, *ECOG* Eastern Cooperative Oncology Group, *Ig* immunoglobulin, *ASCT* autologous stem cell transplantation, *PI* proteasome inhibitor, *IMiD* immunomodulatory drug

^a^Patients from Japan, Korea, and Taiwan

^b^Cytogenetic abnormalities were identified by FISH or karyotype testing. Percentages were calculated with the number of subjects in each treatment group as the denominator

Among East Asian patients, 22 (43.1%) patients in the DRd group and 32 (72.7%) patients in the Rd group discontinued treatment. The most common reasons for discontinuation for DRd vs. Rd were progressive disease (27.5 vs. 63.6%) and adverse events (9.8 vs. 2.3%). Among Japanese patients, 7 (35.0%) patients in the DRd group and 11 (73.3%) patients in the Rd group discontinued treatment. The most common reasons for discontinuation for DRd vs. Rd were progressive disease (15.0 vs. 66.7%) and adverse events (10.0 vs. 0.0%).

The median (range) number of prior lines of therapy was 2 (1–6) for East Asian patients and 1 (1–6) for Japanese patients; 7.3% of East Asian patients and 11.1% of Japanese patients had received >3 prior lines of therapy. Prior therapies included proteasome inhibitors (PIs; in 79.2% of East Asian patients and 83.3% of Japanese patients) and immunomodulatory drugs (in 59.4% of East Asian patients and 44.4% of Japanese patients), including lenalidomide (in 5.2% of East Asian patients and 13.9% of Japanese patients). A total of 62.5% of East Asian patients and 50.0% of Japanese patients had undergone autologous stem cell transplantation, and 44.8% of East Asian patients and 44.4% of Japanese patients were refractory to their last line of therapy.

The median (range) number of treatment cycles received in East Asian patients was 23 (2–33) for DRd and 14.5 (1–32) for Rd. Among Japanese patients, the median (range) number of treatment cycles received was 23 (3–27) for DRd and 11 (3–25) for Rd. The median (range) cumulative lenalidomide dose received for East Asian patients was 5,190.0 (150–15,750) mg for DRd and 5,170.0 (70–15,750) mg for Rd; the median (range) cumulative lenalidomide dose received for Japanese patients was 4,927.5 (150–11,615) mg and 4,670.0 (340–12,950) mg, respectively.

### Efficacy

**Fig. 1:**
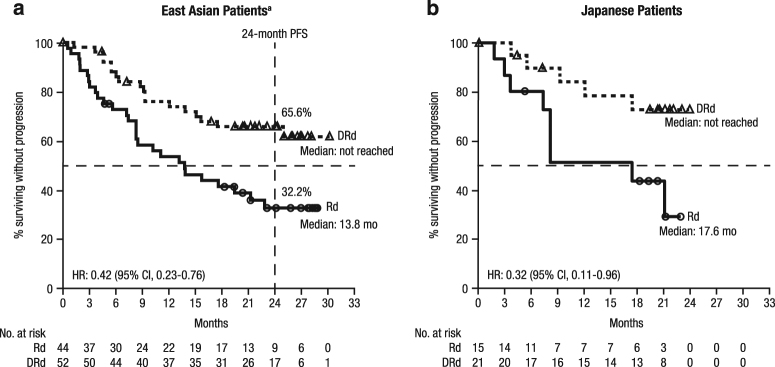
Progression-free survival of East Asian (**a**) and Japanese (**b**) patients in POLLUX. ^a^Patients from Japan, Korea, and Taiwan

**Fig. 2:**
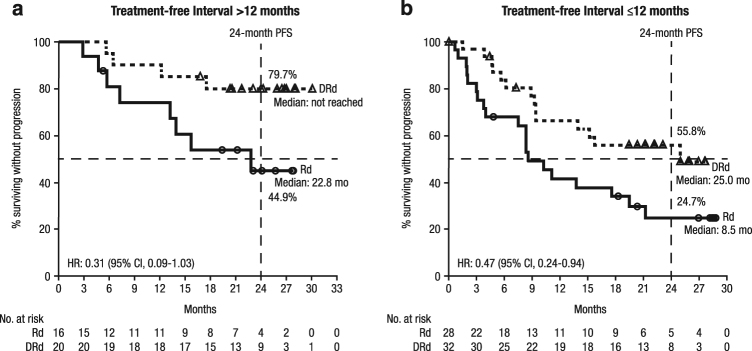
Progression-free survival based on treatment-free interval in East Asian patients in POLLUX. Progression-free survival in East Asian patients with treatment-free intervals of >12 months (**a**) and ≤12 months (**b**).

The median (range) duration of follow-up was 24.7 (0.7–30.5) months in East Asian patients and 21.4 (4.4–24.1) months in Japanese patients. The median PFS for DRd vs. Rd was NR vs. 13.8 months in East Asian patients (HR, 0.42; 95% CI, 0.23–0.76; Fig. 1a) and NR vs. 17.6 months in Japanese patients (HR, 0.32; 95% CI, 0.11–0.96; Fig. 1b). The 24-month PFS rate for DRd vs. Rd was 65.6% (95% CI, 50.5–77.0) vs. 32.2% (95% CI, 18.3–46.9) in East Asian patients; in Japanese patients, the 24-month PFS rate was not estimable (NE) in either treatment group. In patients with a treatment-free interval of >12 months between receipt of last therapy and randomization, the median PFS for DRd vs. Rd was NR vs. 22.8 months (HR, 0.31; 95% CI, 0.09–1.03) in East Asian patients (Fig. 2a). Among these patients, the 24-month PFS rate for DRd vs. Rd was 79.7% (95% CI, 54.5–91.9) vs. 44.9% (95% CI, 18.8–68.1). In East Asian patients with a treatment-free interval of ≤12 months, the median PFS for DRd vs. Rd was 25.0 months vs. 8.5 months (HR, 0.47; 95% CI, 0.24–0.94; Fig. 2b); the 24-month PFS rate for DRd vs. Rd was 55.8% (95% CI, 36.2–71.5) vs. 24.7% (95% CI, 10.1–42.6).

The ORR for DRd vs. Rd was 90.2 vs. 72.1% in East Asian patients and 90.0 vs. 60.0% in Japanese patients (Table 2). Responses to DRd vs. Rd in East Asian patients included 17 (33.3%) vs. 5 (11.6%) stringent complete responses (sCRs), 10 (19.6%) vs. 4 (9.3%) CRs, 11 (21.6%) vs. 8 (18.6%) very good partial responses (VGPRs), and 8 (15.7%) vs. 14 (32.6%) partial responses (PRs). Consistent responses were observed across various subgroups, including those defined by International Staging Sysem staging, cytogenetic risk, number of prior lines of therapy, prior PI exposure, and refractoriness to PIs. Responses to DRd vs. Rd in Japanese patients included 9 (45.0%) vs. 1 (6.7%) sCR(s), 1 (5.0%) vs. 0 (0.0%) CRs, 5 (25.0%) vs. 4 (26.7%) VGPRs, and 3 (15.0%) vs. 4 (26.7%) PRs. The median duration of response for DRd vs. Rd was NE (95% CI, 24.0-NE) vs. 20.2 (95% CI, 12.9-NE) months in East Asian patients and NE (95% CI, NE-NE) vs. 20.2 (95% CI, 2.1-NE) months in Japanese patients. Among responders in the response-evaluable analysis set, the median (range) time to first response for DRd vs. Rd was 1.0 (0.9–13.0) month vs. 1.1 (0.9–8.4) months in East Asian patients and 1.0 (1.0–2.0) months vs. 1.1 (1.0–2.9) months in Japanese patients.

**Table 2:** Overall response rates in the response-evaluable population

	East Asian patients^a^		Japanese patients	
Response, *n* (%)	DRd (*n* = 51)	Rd (*n* = 43)	DRd (*n* = 20)	Rd (*n* = 15)
ORR^b^	46 (90.2)	31 (72.1)	18 (90.0)	9 (60.0)
sCR	17 (33.3)	5 (11.6)	9 (45.0)	1 (6.7)
CR	10 (19.6)	4 (9.3)	1 (5.0)	0 (0.0)
VGPR	11 (21.6)	8 (18.6)	5 (25.0)	4 (26.7)
PR	8 (15.7)	14 (32.6)	3 (15.0)	4 (26.7)
MR	2 (3.9)	3 (7.0)	1 (5.0)	3 (20.0)
SD	3 (5.9)	8 (18.6)	1 (5.0)	3 (20.0)
PD	0 (0.0)	1 (2.3)	0 (0.0)	0 (0.0)
≥CR^c^	27 (52.9)	9 (20.9)	10 (50.0)	1 (6.7)
≥VGPR^d^	38 (74.5)	17 (39.5)	15 (75.0)	5 (33.3)

*DRd* daratumumab/lenalidomide/dexamethasone, *Rd* lenalidomide/dexamethasone, *ORR* overall response rate, *sCR* stringent complete response, *CR* complete response, *VGPR* very good partial response, *PR* partial response, *MR* minimal response, *SD* stable disease, *PD* progressive disease

^a^Patients from Japan, Korea, and Taiwan

^b^sCR + CR + VGPR + PR

^c^sCR + CR

^d^sCR + CR + VGPR

**Fig. 3:**
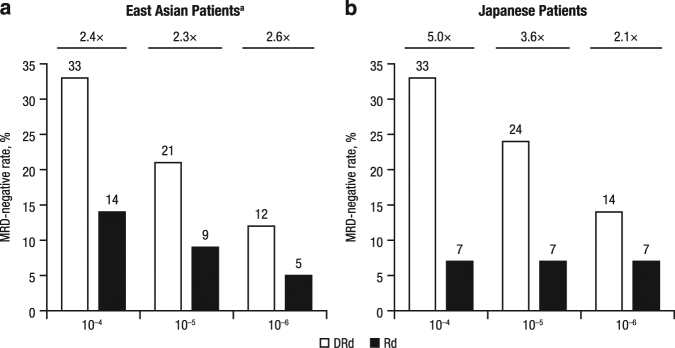
Minimal residual disease–negative rates across three sensitivity thresholds (10^−4^, 10^−5^, 10^−6^) in East Asian (**a**) and Japanese (**b**) patients in the intent-to-treat population in POLLUX. ^a^Patients from Japan, Korea, and Taiwan

Among East Asian patients, MRD-negative rates were higher for DRd vs. Rd (32.7 vs. 13.6% at 10^–4^, 21.2 vs. 9.1% at 10^–5^, and 11.5 vs. 4.5% at 10^–6^; Fig. 3a). Among Japanese patients, MRD-negative rates were also higher for DRd vs. Rd (33.3 vs. 6.7% at 10^–4^, 23.8 vs. 6.7% at 10^–5^, and 14.3 vs. 6.7% at 10^–6^; Fig. 3b).

### Safety

The most common treatment-emergent adverse events (TEAEs; >20% of patients in any group) are listed in Table 3. Consistent with the overall patient population^9^, higher rates of neutropenia, diarrhea, nasopharyngitis, and pyrexia were noted in East Asian patients in the DRd group (35 [68.6%] patients, 21 [41.2%] patients, 20 [39.2%] patients, and 13 [25.5%] patients, respectively) compared with those in the Rd group (21 [47.7%] patients, 8 [18.2%] patients, 12 [27.3%] patients, and 3 [6.8%] patients, respectively). Similar findings were observed in Japanese patients treated with DRd vs. Rd (Table 3).

**Table 3:** Most common (>20%) treatment-emergent adverse events in the safety population

	East Asian patients^a^		Japanese patients	
Event, *n* (%)	DRd (*n* = 51)	Rd (*n* = 44)	DRd (*n* = 20)	Rd (*n* = 15)
Hematologic				
Neutropenia	35 (68.6)	21 (47.7)	13 (65.0)	7 (46.7)
Thrombocytopenia	14 (27.5)	16 (36.4)	3 (15.0)	6 (40.0)
Anemia	12 (23.5)	16 (36.4)	2 (10.0)	2 (13.3)
Leukopenia	10 (19.6)	7 (15.9)	3 (15.0)	3 (20.0)
Lymphopenia	8 (15.7)	8 (18.2)	8 (40.0)	8 (53.3)
Nonhematologic				
Upper respiratory tract infection	23 (45.1)	19 (43.2)	3 (15.0)	3 (20.0)
Constipation	21 (41.2)	14 (31.8)	8 (40.0)	5 (33.3)
Diarrhea	21 (41.2)	8 (18.2)	6 (30.0)	3 (20.0)
Nasopharyngitis	20 (39.2)	12 (27.3)	12 (60.0)	6 (40.0)
Pyrexia	13 (25.5)	3 (6.8)	5 (25.0)	1 (6.7)
Decreased appetite	13 (25.5)	11 (25.0)	1 (5.0)	0 (0.0)
Cough	12 (23.5)	3 (6.8)	0 (0.0)	1 (6.7)
Nausea	11 (21.6)	8 (18.2)	3 (15.0)	0 (0.0)
Insomnia	10 (19.6)	11 (25.0)	2 (10.0)	2 (13.3)
Pneumonia	9 (17.6)	8 (18.2)	2 (10.0)	4 (26.7)
Fatigue	9 (17.6)	10 (22.7)	1 (5.0)	0 (0.0)
Increased alanine aminotransferase	7 (13.7)	4 (9.1)	5 (25.0)	2 (13.3)

*DRd* daratumumab/lenalidomide/dexamethasone, *Rd* lenalidomide/dexamethasone

^a^Patients from Japan, Korea, and Taiwan

The most common grade 3 or 4 TEAEs (>5% of patients in any group) are summarized in Table 4. As expected, the rate of grade 3 or 4 neutropenia was higher in the DRd group compared with the Rd group (34 [66.7%] vs. 19 [43.2%] East Asian patients; 12 [60.0%] vs. 5 [33.3%] Japanese patients). The rate of grade 3 or 4 thrombocytopenia was lower for DRd vs. Rd in both East Asian patients (7 [13.7%] patients vs. 10 [22.7%] patients) and Japanese patients (1 [5.0%] patient vs. 2 [13.3%] patients); these rates were comparable to those in the overall study population (36 [12.7%] patients vs. 38 [13.5%] patients)^9^. The rate of grade 3 or 4 infections for the DRd group vs. the Rd group was 14 (27.5%) vs. 12 (27.3%) East Asian patients and 6 (30.0%) vs. 2 (13.3%) Japanese patients. The most common grade 3 or 4 infection was pneumonia (for DRd vs. Rd, 7 [13.7%] vs. 4 [9.1%] East Asian patients and 2 [10.0%] vs. 2 [13.3%] Japanese patients).

**Table 4:** Most common (>5%) grade 3 or 4 treatment-emergent adverse events in the safety population

	East Asian patients^a^		Japanese patients	
Event, *n* (%)	DRd (*n* = 51)	Rd (*n* = 44)	DRd (*n* = 20)	Rd (*n* = 15)
Hematologic				
Neutropenia	34 (66.7)	19 (43.2)	12 (60.0)	5 (33.3)
Febrile neutropenia	2 (3.9)	2 (4.5)	0 (0.0)	0 (0.0)
Lymphopenia	8 (15.7)	8 (18.2)	8 (40.0)	8 (53.3)
Thrombocytopenia	7 (13.7)	10 (22.7)	1 (5.0)	2 (13.3)
Anemia	6 (11.8)	14 (31.8)	2 (10.0)	2 (13.3)
Leukopenia	5 (9.8)	3 (6.8)	1 (5.0)	0 (0.0)
Leukocytosis	0 (0.0)	1 (2.3)	0 (0.0)	1 (6.7)
Nonhematologic				
Pneumonia	7 (13.7)	4 (9.1)	2 (10.0)	2 (13.3)
Diarrhea	5 (9.8)	2 (4.5)	0 (0.0)	0 (0.0)
Cataract	4 (7.8)	1 (2.3)	2 (10.0)	1 (6.7)
Decreased appetite	4 (7.8)	1 (2.3)	0 (0.0)	0 (0.0)
Increased alanine aminotransferase	3 (5.9)	1 (2.3)	3 (15.0)	0 (0.0)
Hyperglycemia	3 (5.9)	0 (0.0)	2 (10.0)	0 (0.0)
Pyrexia	3 (5.9)	0 (0.0)	0 (0.0)	0 (0.0)
Increased gamma-glutamyltransferase	2 (3.9)	0 (0.0)	2 (10.0)	0 (0.0)
Abnormal hepatic function	2 (3.9)	0 (0.0)	2 (10.0)	0 (0.0)
Hypophosphatemia	2 (3.9)	1 (2.3)	1 (5.0)	1 (6.7)
Somnolence	0 (0.0)	1 (2.3)	0 (0.0)	1 (6.7)
Acute kidney injury	0 (0.0)	1 (2.3)	0 (0.0)	1 (6.7)

*DRd* daratumumab/lenalidomide/dexamethasone, *Rd* lenalidomide/dexamethasone

^a^Patients from Japan, Korea, and Taiwan

Among East Asian patients, serious TEAEs were observed in 26 (51.0%) patients in the DRd group vs. 19 (43.2%) patients in the Rd group; among Japanese patients, they were observed in 10 (50.0%) vs. 4 (26.7%), respectively. The most common serious TEAE in the DRd group was pneumonia, which occurred in 7 (13.7%) East Asian patients treated with DRd and 5 (11.4%) treated with Rd, and in 2 (10.0%) Japanese patients treated with DRd and 1 (6.7%) treated with Rd.

TEAEs led to discontinuation of study treatment in 8 (15.7%) East Asian patients and 3 (15.0%) Japanese patients in the DRd group, and in 2 (4.5%) East Asian patients in the Rd group. No Japanese patients in the Rd group discontinued study treatment due to TEAEs. Among patients in the DRd group who discontinued study treatment due to TEAEs, four East Asian patients (including one Japanese patient) discontinued due to TEAEs possibly or probably related to daratumumab; these TEAEs were grade 3 Epstein-Barr virus-associated lymphoproliferative disorder, grade 3 diarrhea, grade 3 pneumonia, and grade 5 multiple organ dysfunction syndrome. Pneumonia was the most common TEAE leading to discontinuation of study treatment and was observed in 3 (5.9%) East Asian patients and 1 (5.0%) Japanese patient in the DRd group and in 1 (2.3%) East Asian patient and none of the Japanese patients in the Rd group.

In East Asian patients, the median (range) duration of infusion was 7.1 (6.0–14.5) h for the first infusion, 4.4 (3.0–9.4) h for the second infusion, and 3.5 (2.2–6.2) h for all subsequent infusions. In Japanese patients, the median (range) duration of infusion was 7.1 (6.1–14.0) h for the first infusion, 4.4 (4.0–7.5) h for the second infusion, and 3.5 (2.5–4.7) h for all subsequent infusions.

Infusion-related reactions (IRRs) among daratumumab-treated patients occurred in 25 (49.0%) East Asian patients and 7 (35.0%) Japanese patients. Most occurred during the first infusion and were grade 1 or 2 in severity. Grade 3 IRRs occurred in 6 (11.8%) East Asian patients and 1 (5.0%) Japanese patient, and no grade 4 IRRs were observed. The most common IRR was dyspnea, which occurred in 5 (9.8%) East Asian patients and 2 (10.0%) Japanese patients. No patients discontinued treatment due to IRRs.

In the overall POLLUX population, the rates of second primary malignancies were low and were balanced between the two treatment groups (6% in both treatment groups)^18^. Among patients in the East Asian and Japanese subgroups, second primary malignancies were reported in 3 patients in the DRd group: 1 Korean patient (right flank skin site metastatic adenocarcinoma) and 2 Japanese patients (worsening of Bowen’s disease in 1 patient and EBV-positive lymphoproliferative disorder in another). No second primary malignancies were reported in patients in the Rd group.

Among East Asian patients, 10 (19.6%) patients in the DRd group received a total of 49 blood transfusions (8 [15.7%] patients received a total of 26 packed red blood cell transfusions and 5 [9.8%] patients received a total of 23 platelet transfusions), and 12 (27.3%) patients in the Rd group received a total of 57 blood transfusions (12 [27.3%] patients received a total of 48 packed red blood cell transfusions, 1 [2.3%] patient received 2 fresh frozen plasma transfusions, and 5 [11.4%] patients received a total of 7 platelet transfusions). Among Japanese patients, 2 (10.0%) patients in the DRd group received a total of 10 blood transfusions (all were packed red blood cell transfusions), and 2 (13.3%) patients in the Rd group received a total of 8 blood transfusions (2 [13.3%] patients received a total of 6 packed red blood cell transfusions and 1 [6.7%] patient received 2 platelet transfusions).

## Discussion

Consistent with the primary results of the POLLUX study, the addition of daratumumab to Rd significantly reduced the risk of progression/death and increased the rate of deeper responses while demonstrating a favorable safety profile in East Asian patients based on longer follow-up^9^. The hazard ratio for disease progression or death in the daratumumab group vs. control group in these patients was comparable to that of the international population in POLLUX^9^.

In the East Asian subgroup described here, median PFS was NR in the DRd group. These data compare favorably with a subanalysis of Japanese patients in the phase 3 ELOQUENT-2 study of elotuzumab plus Rd (ERd) in patients with RRMM^19^. In the subanalysis of the ELOQUENT-2 study, median PFS was 22.2 months in the ERd arm compared with 18.5 months in the Rd arm^19^. At a milestone of 24 months, the PFS rate of 66% for DRd in East Asian patients in POLLUX compares favorably with the rate of 48% at the same time point in Japanese patients treated with ERd in ELOQUENT-2, although a direct comparison between these two studies should be interpreted with caution due to differences in study design and eligibility criteria^19^.

While the ≥CR rates for DRd among East Asian and Japanese patients were similar to that of the overall POLLUX population, the sCR rates were numerically higher (33.3 and 45.0% in East Asian and Japanese patients, respectively, compared with 26% in the overall POLLUX population)^18^. For the first time in a study of East Asian and Japanese patients with RRMM, a prospective analysis of MRD was performed. At the sensitivity threshold of 10^–5^ for MRD recommended by the IMWG for NGS-based assays^20^, MRD-negative rates in both subgroups were similar to the rates observed for the DRd and Rd treatment groups in the overall POLLUX population^9^.

No new safety signals for the combination of daratumumab and Rd were observed^9^. Neutropenia, a known lenalidomide-associated toxicity, was also the most frequently observed adverse event associated with DRd in these subgroups of patients, consistent with the findings in the overall POLLUX population^9^. Although patients in the DRd group received more treatment cycles compared with those in the Rd group in both subpopulations (East Asian: median of 23.0 vs. 14.5; Japanese: median of 23.0 vs. 11.0), the cumulative doses of lenalidomide received were similar between the treatment groups. Whether the increased neutropenia rate in the DRd treatment group is due to the longer exposure to lenalidomide or due to the introduction of daratumumab remains unclear, and analyses are ongoing based on longer follow-up.

These findings further support the results of other studies examining the efficacy and safety profile of daratumumab in Japanese patients, including the phase 1 MMY1002 study of daratumumab monotherapy in Japanese patients with RRMM^21^ and the phase 1 MMY1005 study of daratumumab plus bortezomib and dexamethasone in Japanese patients with RRMM^22^. Taken together, these studies confirm that Japanese patients derive a similar magnitude of clinical benefit with daratumumab-based regimens compared with patient populations across many other regions and that no new safety signals related to daratumumab can be identified in Japanese or East Asian patients^19^.

There are several limitations to the current study. First, the number of patients in each subgroup, especially in the Japanese subgroup, was low. Second, this was a post hoc rather than a prespecified analysis, so no statistical testing was performed. Third, the impact of this regimen on OS has not yet been determined, as these data were immature at the time of this analysis; follow-up is ongoing.

In summary, the addition of daratumumab to Rd led to prolonged PFS with increased and deeper responses compared with Rd alone in both East Asian patients and Japanese patients from POLLUX, consistent with findings in the international POLLUX population. Given the recent approval of daratumumab in combination with lenalidomide and dexamethasone as a new treatment option in Japan^13^, these findings suggest that this regimen is a new standard of care for Japanese patients with RRMM.

## References

[1] de Weers M (2011). Daratumumab, a novel therapeutic human CD38 monoclonal antibody, induces killing of multiple myeloma and other hematological tumors. J. Immunol..

[2] Lammerts van Bueren, J., et al. Direct in vitro comparison of daratumumab with surrogate analogs of CD38 antibodies MOR03087, SAR650984 and Ab79. *Blood* **124**, Abstract 3474 (2014).

[3] Overdijk MB (2015). Antibody-mediated phagocytosis contributes to the anti-tumor activity of the therapeutic antibody daratumumab in lymphoma and multiple myeloma. mAbs.

[4] van de Donk NWCJ (2016). Monoclonal antibodies targeting CD38 in hematological malignancies and beyond. Immunol. Rev..

[5] Krejcik J (2016). Daratumumab depletes CD38^+^ immune-regulatory cells, promotes T-cell expansion, and skews T-cell repertoire in multiple myeloma. Blood.

[6] Overdijk MB (2016). The therapeutic CD38 monoclonal antibody daratumumab induces programmed cell death via Fcgamma receptor-mediated cross-linking. J. Immunol..

[7] Lokhorst HM (2015). Targeting CD38 with daratumumab monotherapy in multiple myeloma. N. Engl. J. Med..

[8] Lonial S (2016). Daratumumab monotherapy in patients with treatment-refractory multiple myeloma (SIRIUS): an open-label, randomised, phase 2 trial. Lancet.

[9] Dimopoulos MA (2016). Daratumumab, lenalidomide, and dexamethasone for multiple myeloma. N. Engl. J. Med..

[10] Palumbo A (2016). Daratumumab, bortezomib, and dexamethasone for multiple myeloma. N. Engl. J. Med..

[11] Chari A (2017). Daratumumab plus pomalidomide and dexamethasone in relapsed and/or refractory multiple myeloma. Blood.

[12] *DARZALEX*® *(Daratumumab) Injection, for Intravenous Use [Package Insert]*. Janssen Biotech, Inc., Horsham, PA, 2017.

[13] Genmab announces approval of DARZALEX® (daratumumab) for relapsed or refractory multiple myeloma in Japan [news release]. Copenhagen, Denmark: Genmab A/S. September 27, 2017. http://ir.genmab.com/releasedetail.cfm?releaseid=1041758. Accessed September 29, 2017.

[14] Ohtsu A (2011). Bevacizumab in combination with chemotherapy as first-line therapy in advanced gastric cancer: a randomized, double-blind, placebo-controlled phase III study. J. Clin. Oncol..

[15] Kiyota N (2015). Subgroup analysis of Japanese patients in a phase 3 study of lenvatinib in radioiodine-refractory differentiated thyroid cancer. Cancer Sci..

[16] Rajkumar SV (2011). Consensus recommendations for the uniform reporting of clinical trials: report of the International Myeloma Workshop Consensus Panel 1. Blood.

[17] Durie BGM (2006). International uniform response criteria for multiple myeloma. Leukemia.

[18] Bahlis, N., et al. Daratumumab, lenalidomide, and dexamethasone (DRd) vs. lenalidomide and dexamethasone (Rd) in relapsed or refractory multiple myeloma (RRMM): efficacy and safety update (POLLUX). *J. Clin. Oncol.* **35**, Abstract 8025 (2017).

[19] Suzuki K (2017). Randomized phase 3 study of elotuzumab for relapsed or refractory multiple myeloma: ELOQUENT-2 Japanese patient subanalysis. Blood Cancer J..

[20] Kumar S (2016). International Myeloma Working Group consensus criteria for response and minimal residual disease assessment in multiple myeloma. Lancet Oncol..

[21] Iida S (2017). Safety and efficacy of daratumumab in Japanese patients with relapsed or refractory multiple myeloma: a multicenter, phase 1, dose-escalation study. Int. J. Hematol..

[22] Ichinohe, T., et al. Daratumumab with bortezomib+dexamethasone in Japanese pts with relapsed/refractory multiple myeloma. Presented at the 79th Annual Meeting of the Japanese Society of Hematology (JSH); Abstract OS3-12D-3 (Tokyo, 20–22 October 2017).

